# Supplementary material for: Heat adaptation of phage T7 under an extended genetic code
Source: Virus Evol. 2021 Dec 1;7(2):veab100. doi: 10.1093/ve/veab100 (PMC8923235; doi:10.1093/ve/veab100)
Supplement: veab100_Supp [file veab100_supp.zip › Sequencing_Analysis.zip!/Sequencing_Analysis/S1/evidence/RA_32.html]

BRESEQ :: Evidence


| Read alignment evidence... | | | | | | | | | | | |
| --- | --- | --- | --- | --- | --- | --- | --- | --- | --- | --- | --- |
|  | seq id | position | | ref | new | freq | score (cons/poly) | reads | annotation | genes | product |
| \* | NC\_001604.1 | 35,266 | 0 | A | G | 33.3% | 3896.1 / inf | 4112 | intergenic (–/–) | *–/–* | –/– |
| Reads supporting (aligned to +/- strand):  **ref** base A (1532/1189);  **new** base G (678/678);  **total** (2233/1879) | | | | | | | | | | | |
| Fisher's exact test for biased strand distribution *p*-value = 1.62e-04 | | | | | | | | | | | |
| Kolmogorov-Smirnov test that lower quality scores support variant *p*-value = 9.78e-01 | | | | | | | | | | | |

Only 100 of 4195 total aligned reads displayed.  

`ACCTCACTACGGATACTATCGGTGTCAATAACGATGGTCACTTGGATGCTCGTGGTCGTCGAATTGTGAACCTAGCGAACGCCGTGGATGACCGCGATGCTGTTCCGTTTGGTCAACTAAAGACCATGAACCAGAACTCATGGCAAGCACGTAATGAAGCCTTACAGTTCCGTAATGAGGCTGAGACTTTCAGAAACCAAGCGGAGGGCTTTAAGAACGAGTCCAGTACCAACGCTACGAACACAAAGCAGTGGCGCGATGAGACCAAGGGTTTCCGAGACGAAGCCAAGCGGTTCAAGAATACGGCTGGTCAATACGCTACATCTGCTGGGAACTCTGCTTCCGCTGCGCATCAATCTGAGGTAAACGCTGAGAACTCT‑‑GCCACAGCATCCGCTAACTCTGCTCATTTGGCAGAACAGCAAGCAGACCGTGCGGAACGTGAGGCAGACAAGCTGGAAAATTACAATGGATTGGCTGGTGCAATTGATAAGGTAGATGGAACCAATGTGTACTGGAAAGGAAATATTCACGCTAACGGGCGCCTTTACATGACCACAAACGGTTTTGACTGTGGCCAGTATCAACAGTTCTTTGGTGGTG  >  NC_001604.1/34967‑35566`  

                                                                                                                                                                                                                                                                                                           `|`                                                                                                                                                                                                                                                                                                                
`accacgcTACGGATACTATCGGTTTCAATGACGCTGGTCACTTGGATGCTCGTGGTCGTCGAATTGTGAACCTAGCGAACGCCGAAGATGACCGCGATGCTGTTCCGTTTGGTCAACTAAAGACCATGGACCAGAACTCATGGCTAGCACGTAATGAAGCCTTACAGTTCCGTAATGAGGCTGAGACTTTCAGAAACCAAGCGGAGGGCTTTAAGAACGAGTCCAGTACCAACGCTACGAACACAAAGCAGTGGCGCGATGAGACCAAGGGTTTCCGAGACGAAGCCAAGCGGTTCAAGaa                                                                                                                                                                                                                                                                                                               <  1:615868/295‑1 (MQ=255)`  
`aaatCACTACGTATAGTATCGGTGTCAATAACGAAGGGCAGTTGGATGCACGTGGTCGTCGAATCTTTAACTAAGCGAACGCCGAGGAAGGCTGGGATGCTGTGCAGTTTGGTCAACTAAGGACCATGTACCAGAACTGATGGCAAGGACGTAATGAAGCCTAACAGTTCGGAAATGAGGATGAGACTTTCAGAAACAAAGCGGAGGGCTTTAAGAACGGGTCCAGTACCAACGCTACGAACACAAAGCAGTGGCGCGATGAGACCAAGGGTTTCCGAGACGAAGCCAAGCGGTTCAAGaa                                                                                                                                                                                                                                                                                                               <  1:479768/298‑1 (MQ=255)`  
 `ccTCACTACGGATACTATCGGTGTCAATAACGATGGTCACTTGGATGCTCGTGGTCGTCGAATTGTGAACCTAGCGAACGCCGTGGATGACCGCGATGCTGTTCCGTTTGGTCAACTAAAGACCATGGACCAGAACTCATGGCAAGCACGTAATGAAGCCTTACAGTTCCGTAATGAGGCTGAGACTTTCAGAAACCAAGCGGAGGGCTTTAAGAACGAGTCCAGTACCAACGCTACGAACACAAAGCAGTGGCGCGATGAGACCAAGGGTTTCCGAGACGAAGCCAAGCGGTTCAAGaaa                                                                                                                                                                                                                                                                                                              >  1:331605/1‑300 (MQ=255)`  
 `aagagacagggATACTATCGGTGTCAATGACGATGGTCACTTGGATGCTCGTGGTCGTCGAATTGTGAACCTAGCGAACGCCGTGGATGACCGCGATGCTGTTCCGTTTGGTCAACTAAAGACCAGGGACCAGAACTCATGGCAAGCACGTAATGAAGCCTTACAGTTCCGTAATGAGGCTAAGACTTTCAGAAACCAAGCGGAGGGCTTTAAGAACGAGTCCAGTACCAACGCTACGAACACAAAGCAGTGGCGCGATGAGACCAAGGGTTTCCTAGACGAAGCCAAGCGGTTCAAGGAt                                                                                                                                                                                                                                                                                                              <  1:198585/292‑1 (MQ=255)`  
   `tCACTACGGCTACTATCGGTGTCAATAACGATGGTCACTTGGATGCTCGTGGTCGTCTAATTGTGAACCTAGCGAACGCCGTGGATGACCGCGATGCTGTTCCGTTTGGTCAACTAAAGACCATGGACCAGAACTCATGGCAAGCACGTAATGAAGCCTTACAGTTCCGTAATGAGGCTGAGACTTTCAGAAACCAAGCGGAGGGCTTTAAGAACGAGTCCAGTACCAACGCTACGAACACAAAGCAGTGGCGCGATGAGACCAAGGGTTTCCGAGACGAAGCCAAGCGGTTCAAGGATAc                                                                                                                                                                                                                                                                                                            <  1:627338/301‑1 (MQ=255)`  
   `tCACTACGGATACTATCGTTGTAGATATCGATGTTCACTTGGATGCTCGTGGTCGTCGAATTGTGAACCTAGCGAACGCCGGGGATGACCGCGATTCTGTTCCGTTTGGTCAACTAAAGACCATCTACCAGAACTCATTGCAAGCACGTAATGCAGCCTTACAGTTCAGTAATGCGTGTGAGACTTTCAGAAACCAAGCGGAGGGCTTTAAGAACAAGTCCAGTACCATCTCTACGAACACTAAGCAGTGGCGCGATGAGACCATGAGTTTCTGAGACGAAGCCAAGCGGTTCAAGGATAc                                                                                                                                                                                                                                                                                                            <  1:653483/301‑1 (MQ=255)`  
   `ggggtcggagatgtgaagaagagacggaCGATGGTCACTTGGATGCTGGGGGTCGTCGAATTGTGAACCTAGCGAACGCCGTGGATGACCGCGATGCTGTTCCGTTTGGTCAACTAAAGACCATGGACCAGAACTCATGGCAAGCACGTAATGAAGCCTGACAGTTCCGTAATGAGGCTGAGACTTTCAGAAACCAAGCGGAGGGCTTTAAGAACGAGTCCAGTACCAACGCTACGAACACAAAGCAGTGGCGCGATGAGACCAAGGGTTTCCGAGACGAAGCCAAGCGGTTCAAGGATAc                                                                                                                                                                                                                                                                                                            <  1:17401/274‑1 (MQ=255)`  
   `gacagaCGGATACTATCGGTGTCAATAACGATGGTCACTTGGATGCTCGTGGTCGTCGAATTGTGAACCTAGCGAACGCCGTGGATGACCGCGATGCTGTTCCGTTTGGTCAACTAAAGACCATGGACCAGAACTCATGGCAAGCACGTAATGAAGCCTTACAGTTCCGTAATGAGGCTGAGACTTTTAGAAACCAAGCGGAGGGCTTTAAGAACGAGTCCAGTACCAACGCTACGAACACAAAGCAGTGGCGCGATGAGACCAAGGGTTTCCGAGACGAAGCCAAGCGGTTCAAGGATAc                                                                                                                                                                                                                                                                                                            <  1:341825/296‑1 (MQ=255)`  
   `ccACTACGGATACTATCGGGGTCAAGAACGATGGTCACTTGGATGCTCGTGGTCGGCGAATTGTGAACCTAGCGAACGCCGTGGAGGACCGCGATGCTGTTCCGTTTGGTCAACTAAAGACCATGGACCAGAACTCATGGCAAGCACGTAATGAAGCCTTACAGTTCCGTAATGAGGCTGAGACTTTCAGAAACCAAGCGGAGGGCTTTAAGAACGAGTCCAGTACCAACGCTACGAACACAAAGCAGTGGCGCGATGAGACCAAGGGTTTCCGAGACGAAGCCAAGCGGTTCAAGAATAc                                                                                                                                                                                                                                                                                                            <  1:486123/300‑1 (MQ=255)`  
   `agagacaggATACGATCGGTGTCAATAACGATGTGCACTTGGATGCTCGTGGTCGGCGAATTGTGAACCTAGCGAACGCCGTGGATGACCGCGATGCTGTTCCGTTTGGTCAACTAAAGACCATGGACCAGAACTCATGGCAAGCACGTAATGAAGCCTTACAGTTCCGTAATGAGGCTGAGACTTTCAGAAACCAAGCGGAGGGCTTTAAGAACGAGTCCAGTACCAACGCTACGAACACAAAGCAGTGGCGCGATGAGACCAAGGGTTTCCGAGACGAAGCCAAGCGGTTCAAGGATAc                                                                                                                                                                                                                                                                                                            <  1:622294/294‑1 (MQ=255)`  
   `agacagcGGATACTAGCGGTGTAAATAACGATGGTCACTTGGATGCTCGTGGACGTCGAATGGGGAACCTAGCGAACGCCGTGGATGACCGCGATGCTGTTCCGTGTGGTCAACTAAAGAGCATGGACCAGAACTCATGGCAAGCACGTAATGAAGCCTAACAGTTCCGTAATGAGGCTGAGACTTTCAGAAACCAAGCGGAGGGCTTTAAGAACGAGTCCAGTACCAACGCGACGAACACAAAGCAGTGGCGCGATGAGACCAAGGGTTTCCGAGACGAAGCCAAGCGGTTCAAGGATAc                                                                                                                                                                                                                                                                                                            <  1:532176/295‑1 (MQ=255)`  
    `cACTACGGATACTATCGGTGTCAATAACGATGGTCACTTGGATGCTCGTGGTCGTCGAATTGTGAACCTAGCGAACGCCGTGGATGACCGCGATGCTGTTCCGTTTGGTCAACTAAAGACCATGGACCAGAACTCATGGCAAGCACGTAATGAAGCCTTACAGTTCCGTAATGAGGCTGAGACTTTCAGAAACCAAGCGGAGGGCTTTAAGAACGAGTCCAGTACCAACGCTACGAACACAAAGCAGTGGCGCGATGAGACCAAGGGTTTCCGCGACGAAGCCAAGCGGTTCAAGAATACg                                                                                                                                                                                                                                                                                                           >  1:588168/1‑301 (MQ=255)`  
      `cTACGGATACTATCGGTGTCAATAACGATGGTCACTTGGATGCTCGTGGTCGTCGAATTGTGAACCTAGCGAACGCCGTGGATGACCGCGATGCTGTTCCGTTTGGTCAACTAAAGACCATGGACCAGAACTCATGGCAAGCACGTAATGAAGCCTTACAGTTCCGTAATGAGGCTGAGACTTTCAGAAACCAAGCGGAGGGCTTTAAGAACGAGTCCAGTACCAACGCTACGAACACAAAGCAGTGGCGCGATGAGACCAAGGGTTTCCGAGACGAAGCCAAGCGGTTCAAGaactgtct                                                                                                                                                                                                                                                                                                         >  1:566007/1‑295 (MQ=255)`  
      `atACGGATACTATCGGTGTCAATAACGATGGTCACTTGGATGCTCGTGGTCGTCGAATTGTGAACCTAGCGAACGCCGTGGATGACCGCGATGCTGTTCCGTTTGGTCAACTAAAGACCATGGACCAGAACTCATGGCAAGCACGTAATGAAGCCTTACAGTTCCGGAATGAGGCTGAGACTTTCAGAAACCAAGCGGAGGGCTTTAAGAACGAGACCAGTACCAACGCTACGAACACAAAGCAGTGGCGCGATGAGACCAAGGGTTTCCGAGACGAAGCCAAGCGGTTCAAGAATACGGc                                                                                                                                                                                                                                                                                                         <  1:555648/300‑1 (MQ=255)`  
      `agaCGGATACTATCGGTGTCAATAACGATGGTCACTTGGATGCTCGTGGTCGTCGAATTGTGAACCTAGCGAACGCCGTGGATGACCGCGATGCTGTTCCGTTTGGTCAACTAAAGACCATGGACCAGAACTCATGGCAAGCACGTAATGAAGCCTTACAGTTCCGTAATGAGGCTGAGACTTTCAGAAACCAAGCGGAGGGCTTTAAGAACGAGTCCAGTACCAACGCTACGAACACAAAGCAGTGGCGCGATGAGACCAAGGGTTTCCGAGACGAAGCCAAGCGGTTCAAGGATACGGc                                                                                                                                                                                                                                                                                                         <  1:111711/299‑1 (MQ=255)`  
       `gaCGGATACTATTGGTGTCAATAACGATGGTCACTTGGATGCTCGTGGTCGTCGAATTGTGAACCGAGCGAACGTCGTGGATGACCGCGATGCTGTTCCGGTTGGTCAACTAAAGACCATGGACCAGAACTCATGGCAAGCACGTAATGAAGCCTTACAGTTCCGTAATGAGGCTGAGACTTTCAGAAACCAAGCGGAGGGCTTTAAGAACGAGTCCAGTACCAACGCTACGAACACAAAGCAGTGGCGCGATGAGACCAAGGGTTTCCGAGACGAAGCCAAGCGGTTCAAGAATACGGCt                                                                                                                                                                                                                                                                                                        <  1:69222/300‑1 (MQ=255)`  
           `gATACTATCGGTGTCAATAACGATGGTCACTTGGATGCTCGTGGTCGTCGAATTGTGAACCTAGCGAACGCCGTGGATGACCGCGATGCTGTTCCGTTTGGTCCACTAAAGACCATGGACCAGAACTCATGGCAAGCACGTAATGAAGCCTTACAGTTCCGTAATGAGGCTGAGACTTTCAGAAACCAAGCGGAGGGCTTTAAGAACGAGTCCAGTACCAACGCTACGAACACAAAGCAGTGGCGCGATGAGACCAAGGGTTTCCGAGACGAAGCCAAGCGGTTCAAGGATACGGCTgggc                                                                                                                                                                                                                                                                                                    >  1:571318/1‑299 (MQ=255)`  
               `cTATCGGTGTCAATAACGATGGTCACTTGGATGCTCGTGGTCGTCGAATTGTGAACCTAGCGAACGCCGTGGATGACCGCGATGCTGTTCCGTTTGGTCAACTAAAGACCATGGACCAGAACTCATGGCAAGCACGTAATGAAGCCTTACAGTTCCGTAATGAGGCTTAGACTTTCAGAAACCAAGCGGAGGGCTTTAAGAACGAGTCCAGTACCAACGCTACGAACACAAAGCAGTGGCGCGATGAGACCAAGGGTTTCCGAGACGAAGCCAAGCGGTTCAAGAATACGGCTGGTAAATa                                                                                                                                                                                                                                                                                                >  1:426485/1‑301 (MQ=255)`  
               `cTATCGGTGTCAATAACGATGGTCACTTGGATGCTCGTGGTCGTCGAATTGTGAACCTAGCGAACGCCGTGGATGACCGCGATGCTGTTCCGTTTGGTCAACTAAAGACCATGGACCAGAACTCATGGCAAGCACGTAATGAAGCCTTACAGTTCCGTAATGAGGCTGAGACTTTCAGAAACCAAGCGGAGGGCTTTAAGAACGAGTCCAGTACCAACGCTACGAACACAAAGCAGTGGCGCGATGAGACCAAGGGTTTCCGAGACGAAGCCAAGCGGTTCAAGAATACGGCTGGTCAATa                                                                                                                                                                                                                                                                                                >  1:561080/1‑301 (MQ=255)`  
               `cTATCGGTGTCAATAACGATGGTCACTTGGATGCTCGTGGTCGTCGAATGGTGAACCTAGCGAACGCCGTGGATGACCGCGATGCTGTTGCGTTTGGTCAACTAAAGACCATGGACCAGAACTCATGGCAAGAACGGAATGAAGCCTTACAGTGCCGTAATGAGGCTGAGACTTTCAGAAACCAAGCGGAGGGCTTTAAGAACGAGTCAAGTACCAACGCTACGAACACAAAGCAGTGGCGCGATGAGACCAAGGGTTTCCGAGACGAAGCCAAGCGGGTCAAGGATACGGAGGGTCAATa                                                                                                                                                                                                                                                                                                >  1:270991/1‑301 (MQ=255)`  
                `tATCGTTGTCGATAACGATGGTCACTTGGATGCTCGTGGTCGTCGAATTGTGAACCTAGCGAACGACGTGGATGACCGCGATGCTGTTCCGTTTGGTCAACTAAAGACCATGGACCAGAACTCATGGCAAGCACGTAATGAAGCCTTACAGTTCCGTAATGAGGCTGAGACTTTCAGAAACCAAGCGGAGGGCTTTAAGAACGAGTCCAGTACCAACGCTACGAACACAAAGCAGTGGCGCGATGAGACCAAGGGTTTCCGAGACGAAGCCAAGCGGTTCAAGAATACGGCTGGTCAATAc                                                                                                                                                                                                                                                                                               <  1:544151/301‑1 (MQ=255)`  
                 `aTCGGTGTCAATAACGATGGTCACTTGGATGCTCGTGGTCGTCGAATTGTGAACCTAGCGAACGCCGTGGATGACCGCGATGCTGTTCCGTTTGGTTAACTAAAGACCATGGACCAGAACTCATGGCAAGCACGTAATGAAGCCTTACAGTTCCGTAATGAGGCTGAGACTTTCAGAAACCAAGCGGAGGGCTTTAAGAACGAGTCCAGTACCAACGCTACGAACACAAAGCAGTGGCGCGATGAGACCAAGGGTTTCCGAGACGAAGCCAAGCGGTTCAAGAATACGGCTGGTCAATACg                                                                                                                                                                                                                                                                                              >  1:577353/1‑301 (MQ=255)`  
                  `tCGGTGTCAATAACGATGGTCACTTGGATGCTCGTGGTCGTCGAATTGTGAACCTAGCGAACGCCGTGGATGACCGCGATGCTGTTCCGCTTGGTCAACTAAAGACCATGGACCAGAACTCATGGCAAGCACGTAATGAAGCCTTACAGTTCCGTAATGAGGCTGAGACTTTCAGAAACCAAGCGGAGGGCTTTAAGAACGAGTCCAGTACCAACGCTACGAACACAAAGCAGTGGCGCGATGAGACCAAGGGTTTCCGAGACGAAGCCGAGCGGTTCAAGAATACGGCTGGTCAATACGc                                                                                                                                                                                                                                                                                             <  1:186234/301‑1 (MQ=255)`  
                        `gcAATAACGATGGTCACTTGGATGCTCGTGGTCGTCGAATTGTGAACCTAGCGAACGCCGTGGATGACCGCGATGCTGTTCCGTTTGGTCAACTAAAGACCATGGACCAGAACTCATGGCAAGCACGTAATGAAGCCTTACAGTTCCGTAATGAGGCTGAGACCTTCAGAAACCAAGCGGAGGGCTTTAAGAACGAGTCCAGTACCAACGCTACGAACACAAAGCAGTGGCGCGATGAGACCAAGGGTTTCCGAGACGAAGCCAAGCGGTTCAAGGATACGGCTGGTCAATACGCTACATc                                                                                                                                                                                                                                                                                       <  1:287282/300‑1 (MQ=255)`  
                         `cAATAACGATGGTCACTTGGATGCTCGTGGTCGTCGAATTGTGAACCTAGCGAACGCCGTGGATGACCGCGATGCTGTTCCGTTTGGTCAACTAAAGACCATGGACCAGAACTCATGGCAAGCACGTAATGAAGCCTTACAGTTCCGTAATGAGGCTGTGGCTTTCAGAAACCAAGCGGAGGGCTTTAAGAACGAGTCCAGTACCAACGCTACGAACACAAAGCAGTGGCGCGATGAGACCAAGGGTTTCCGAGACGAAGCCAAGCGGTTCAAGAATACGGCTGGCCAATACGCAACAtac                                                                                                                                                                                                                                                                                      >  1:439166/1‑299 (MQ=255)`  
                         `cAATAACGATGGTCACTTGGATGCTCGTGGTCGTCGAATTGTGAACCTAGCGAACGCCGTGGATGACCGCGATGCTGTTCCGTTTGGTCAACTAAAGACCATGGACCAGAACTCATGGCAAGCACGTAATGAAGCCTTACAGTTCCGTAATGAGGCTGAGACTTTCAGAAACCAAGCGGAGGGCTTTAAGAACGAGTCCAGTACCAACGCTACGAACACAAAGCAGTGGCGCGATGAGACCAAGGGTTTCCGAGACGAAGCCAAGCGGTTCAAGAATACGGCTGGTCAATACGCTACATcc                                                                                                                                                                                                                                                                                      >  1:86366/1‑300 (MQ=255)`  
                          `aaTAACGATGGTCACTTGGATGCTCGTGGTCGTCGAATTGTGAACCTAGCGAACGCCGTGGATGACCGCGATGCTGTTCCGTTTGGTCAACTAAAGACCATGGACCAGAACTCATGGCAAGCACGTAATGAAGCCTTACAGTTCCGTAATGAGGCTGAGACTTTCAGAAACCAAGCGGAGGGCTTTAAGAACGAGTCCAGTACCAACGCTACGAACACAAAGCAGTGGCGCGATGAGACCAAGGGTTTCCGAGACGAAGCCAAGCGGTTCAAGAATACGGCTGGTCAATACGCTACATctg                                                                                                                                                                                                                                                                                     <  1:156669/301‑1 (MQ=255)`  
                                  `acagcACTTGGATGTTAGTGGTAGTCGAATTGGGAACCTAGCGAACGCCGTGGATGACCGCGATGCTGTTCTGTTTGGTCAACTAAAGACCATGGACAAGAACTCATGGAAAGCACGTAATGAAGCCTTACAGTTCCGTAGAGAGGCTGAGACTTTCAGAAGCCAAGCGGATGGCTTTAAGAACGAGTCCAGTACCAACGCTACGAACAGAAAGCAGTGGCGCGATGAGACCAAGGGTTTCCGAGACGAAGCCAAGCGGTTCAAGAATACGGCTGGTCAATACGCTACATCTGCTGGGAAc                                                                                                                                                                                                                                                                             <  1:401448/297‑1 (MQ=255)`  
                                   `ggTCACTTGGATGCTCGTGGTCGTCGAATTGTGAACCTAGCGAACGCCGTGGATGACCGCGATGCTGTTCCGTTTGGTCAACTAAAGACCATGGACCAGAACTCATGGCAAGCACGTAATGAAGCCTTACAGTTCCGTAATGAGGCTGAGACTTTCAGAAACCAAGCGGAGGGCTTTAAGAACGAGTCCAGTACCAACGCTACGAACACCAAGCAGTGGCGCGATGAGACCAAGGGTTTCCGAGACGAAGCCAAGCGGTTCAAGAATACGGCTGGTCAATACGCTACATCTGCTGGGAAct                                                                                                                                                                                                                                                                            >  1:178917/1‑301 (MQ=255)`  
                                   `ggTCACTTGGATGCTCGTGGTCGTCGAATTGTGAACCTAGCGAACGCCGTGGATGACCGCGATGCTGTTCCGTTTGGTCAACTAAAGACCATGGACCAGAACTCATGGCAAGCACGTAATGAAGCCTTACAGTTCCGTAATGAGGCTGAGACTTTCAGAAACCAAGCGGAGGGCTTTAAGAACGAGTCCAGTACCAACGCTACGAACACAAAGCAGTGGCGCGATGAGCCCAAGGGTTTCCGAGACGAAGCCAAGCGGTTCAAGAATACGGCTGGTCAATACGCTACATCTGCCGGGAAct                                                                                                                                                                                                                                                                            >  1:503140/1‑301 (MQ=255)`  
                                    `gTCACTTGGATGCTCGTGGTCGTCGAATTGTGAACCTAGCGAACGCCGTGGATGACCGCGATGCTGTTCCGTTTGGTCAACTAAAGACCATGGACCAGAACTCATGGCAAGCACGTAATGAAGCCTTACAGTTCCGTAATGAGGCTGAGACTTTCAGAAACCAAGCGGAGGGCTTTAAGAACGAGTCCAGTACCAACGCTACGAACACAAAGCAGTGGCGCGATGAGACCAAGGGTTTCCGAGACGAAGCCAAGCGGTTCAAGGATACGGCTGGTCAATACTCTACATCTGCTGGGAActc                                                                                                                                                                                                                                                                           >  1:407489/1‑301 (MQ=255)`  
                                                      `gtcgtcGAATTGTGAACCTAGCGAACGCCGTGGATGACCGCGATGCTGTTCCGTTTGGTCAACTAAAGACCATGGACCAGAACTCATGGCAAGCACGTAATGAAGCCTTACAGTTCCGTAATGAGGCTGAGACTTTCAGAAACCAAGCGGAGGTCTTTAAGAACGAGTCCAGTACCAACGCTACGAACACAAAGCAGTGGCGCGATGAGACCAAGGGTTTCCGAGACGAAGCCAAGCGGTTCAATGATACGGCTGGTCAATACGCTACATCTGCTGGGAACTCTGCTTCCGCTGCGCATCa                                                                                                                                                                                                                                                         >  1:610571/1‑301 (MQ=255)`  
                                                         `gtcgAATTGTGAAACTAGCTAAGGACGTGGATGACCGCGATGCTGTTCCGTTTGGGCAACTAAAGACCATGGACCAGAACTCATGGCAAGCACGTAATGAAGCCTTACAGTTCCGTAATGAGGCTGAGACTTTCAGAAACCAAGCGGAGGGCTTTAAGAACGAGTCCAGTACCAACGCTACGAACACAAAGCAGTGGCGCGATGAGACCAAGGGTTTCCGAGACGAAGCCAAGCGGTTCAAGAATACGGCTGGTCAATACGCTACATCTGCTGGGAACTCTGCTTCCGCTGCGCATCAATc                                                                                                                                                                                                                                                      <  1:353178/301‑1 (MQ=255)`  
                                                            `gAATTGTGAACCTAGCGAACGCCGTGGATGACCGCGATGCTGTTCCGTTTGGTCAACTAAAGACCATGGACCAGAACTCATGGCAAGCACGTAATGAAGCCTTACAGTTCCGTAATGAGGCTGAGACTTTCAGAAACCAAGCGGAGGGCTTTAAGAACGAGTCCAGTACCAACGCTACGAACACAAAGCAGTGGCGCGATGAGACCACGGGTTTCCGAGACGAAGCCGAGCGGTTCAAGAATACGGCTAGTCAATACGATACATCTGCTGGGAACTCTGCTTCCGCTGCGCATCCATCtct                                                                                                                                                                                                                                                   >  1:52619/1‑299 (MQ=255)`  
                                                                         `aGCGAACGCCGTGGATGACCGCGATGCTGTTCCGTTTGGTCAACTAAAGACCATGGACCAGAACTCATGGCAAGCACGTAATGAAGCCTTACAGTTCCGTAATGAGGCTGAGACTTTCAGAAACCAAGCGGAGGGCTTTAAGAACGAGTCCAGTACCAACGCTACGAACACAAAGCAGTGGCGCGATGAGACCAAGGGTTTCCGAGACGAAGCCAAGCGGTTCAAGAATACGGCTGGTCAATACGCTACATCTGCTGGGAACTCTGCTTCCGCTGCGCATCAATCTGAGGTAAACGCTgag                                                                                                                                                                                                                                      >  1:347386/1‑301 (MQ=255)`  
                                                                               `cGCCGTGGATGACCGCGATGCTGTTCCGTTTGGTCAACTAAAGACCATGGACCAGAACTCATGGCAAGCACGTAATGAAGCCTTACAGTTCCGTAATGAGGCTGAGACTTTCAGAAACCAAGCGGAGGGCTTTAAGAACGAGTCCAGTACCAACGCTACGAACACAAAGCAGTGGCGCGATGAGACCAAGGGTTTCCGGGACGAAGCCAAGCGGTTCAAGAATACGGCTGGTCAATACGCTACATCTGCTGGGAACTCTGCTTCCACTGCGCATCAATCTGAGGTAAACGCTGAGCActct                                                                                                                                                                                                                                >  1:472648/1‑301 (MQ=255)`  
                                                                                    `aggATGACCGCGAGGCTGTTCCGTTTGGTCAACTAAAGACCAGGGACCAAAACGCATGGCAAGCACGTAATGAAGCCTTACAGTACCGTAATGAGGCTGAGACTTTCAGAAACCAAGCGGAGGGCTTTAAGAACGAGTCCAGTACCAACGCTACGAACACAAAGCAGTGGCGCGATGAGACCAAGGGTTTCCGAGACGAAGCCAAGCGGTTCAAGGATACGGCTGGTCAATACGCTACATCTGCTGGGAACTCTGCTTCCGCTGCGCATCAATCTGAGGTAAACGCTGAGAACTCT‑‑GCcac                                                                                                                                                                                                                         <  1:462481/300‑1 (MQ=255)`  
                                                                                      `gaagagacagATGCGGTTCCGTTTGGTCAACTAAAGACCATGGACAAGAACTCATGGCAAGCACGTAATGAAGCCTTACAGTTCCGTAATGAGGCTGAGACGTTCAGAAACCAAGCGGAGGTCTTTAAGAACGAGTCCAGTCCCAACGCTACGAACACAAAGCAGTGGCGCGATGAGACCAAGGGTTTCCGAGACGAAGCCAAGCGGTTCAAGGATACGGCTGGTCAATACGCTACATCTGCTGGGAACTCTGCTTCCGCTGCGCATCAATCTGAGGTAAACGCTGAGAACTCT‑‑GCCACAg                                                                                                                                                                                                                       <  1:419517/292‑1 (MQ=255)`  
                                                                                       `aTGACCGCGATGCTGTTCCGTTTGGTCAACTAAAGACCATGGACCAGAACTCATGGCAAGCACGTAATGAAGCCTTACAGATCCGTAATGAGGCTGAGACTGTCAGAAACCAAGCGAAGGGCTTTAAGAACGAGTCCAGTACCAACGCTACGAACACAAAGCAGTGGCGCGATGAGACCAAGGGTTTCCGAGACGAAGCCAAGCGGTTCAAGAATACGGCTGGTCAATACGCTACATCTGCTGGGAACTCTGCTTCCGCTGCGCATCAATCTGAGGTAAACGCTGAGAACTCT‑‑GCCACAGc                                                                                                                                                                                                                      <  1:449474/301‑1 (MQ=255)`  
                                                                                                `aTGCTGTTCCGTTTGGTCAACTAAAGACCATGGACCAGAACTCATGGCAAGCACGTAATGAAGCCTTACAGTTCCGTAATGAGGCTGAGACTTTCAGAAACCAAGCGGAGGGCTTTAAGAACGAGTCCAGTACCAACGCTACGAACACAAAGCAGTGGCGCGATGAGACCAAGGGTTTCCGAGACGAAGCCGAGCGGTTCAAGAATACGGCTGGTCAATACGCTACATCTGCTGGGAACTCTGCTTCCGCTGCGCATCAATCTGAGGTAAACGCTGAGAACGCT‑‑GCCACAGCATCCGCaaa                                                                                                                                                                                                             >  1:381578/1‑301 (MQ=255)`  
                                                                                                   `ttgttatgTTTGGTCAACTAAAGACCATGGACCAGAACTCATGGCAAGCACGTAATGAAGCCTTACAGTTCCGTAATGTGGCTGAGACTTTCAGAAACCAAGCGGAGGGCTTTAAGAACGAGTCCAGTACCAACGCTACGAACACAAAGCAGTGGCGCGATGAGACCAAGGGTTTCCGAGACGAAGCCAAGCGGTTCAAGGATACGGCTGGTCAATACGCTACATCTGCTGGGAACTCTGCTTCCGCTGCGCATCAATCTGAGGTAAACGCTGAGAACTCT‑‑GCCACAGCATCCGCTAActa                                                                                                                                                                                                          <  1:204916/294‑2 (MQ=255)`  
                                                                                                   `cTGTTCCGTTTGGTCAACTAAAGACCATGGACAAGAACTCATGGCAAGCACGTAATGAAGCCTTACAGTTCCGTAAGGAGGCTGAGAATTTCAGAAACCAAGCGGAGGGCTTTAAGAACGAGTCCAGTACCAACGCTACGAACACAAAGCAGTGGCGCGATGAGACCAAGGGTGTCCGAGACGAAGCCGAGCGGTTCAAGAATACGGCTGGTCAATACGCTACATCTGCTGGGAACTCTGCTTCCGCTGCGCATCAATCTGAGGTAAACGCTGAGAACTCT‑‑GCCACAGCATCCGCTAActa                                                                                                                                                                                                          <  1:123418/301‑2 (MQ=255)`  
                                                                                                     `gTTCCGTTTGGTCAACTAAAGACCATGGACCAGAACTCATGGCAAGCACGTAATGAAGCCTTACAGTTCCGTAATGAGGCTGAGACTTTCAGAAACCAAGCGGAGTGCTTTAAGAACGAGTCCAGTACCAACGCTACGAACACAAATCAGTGGCGCGATGAGACCAAGGGTTTCCGAGACGAAGCCAAGCGGTTCAAGGATACGGCTGGTCAATACGCTACCTCTGCTGGGAACTCTGCTTCCGCTGCGCATCAATCTGAGGTAAACGCTGAGCACTCT‑‑GCCACCGCCTCCGCTAActttt                                                                                                                                                                                                        >  1:361748/1‑298 (MQ=255)`  
                                                                                                     `gTTCCGTTTGGTCAACTAAAGACCATGGACCAGAACTCATGGCAAGCACGTAATGAAGCCTTACAGTTCCGTAATGAGGCTGAGACTTTCAGAAACCAAGCGGAGGGCTTTAAGAACGAGTCCAGTACCAACGCTACGAACACAAAGCAGTGGCGCGATGAGACCAAGGGTTTCCGAGACGAAGCCAAGCGGTTCAAGGATACGGCTGGTCAATACGCTACATCTGCTGGGAACTCTGCTTCCGCTGCGCATCAATCTGAGGTAAACGCTGAGAACTCT‑‑GCCACAGCATCCGCTAActttt                                                                                                                                                                                                        >  1:210544/1‑298 (MQ=255)`  
                                                                                                                   `aCTAAAGACCATGGACCAGAACTCATGGCAAGCACGTAATGAAGCCTTACAGTTCCGTAATGAGGCTGAGACTTTCAGAAACCAAGCGGAGGGCTTTAAGAACGAGTCCAGTACCAACGCTACGAACACAAAGCAGTGGCGCGATGAGACCAAGGGTTTCCGAGACGAAGCCAAGCGGTTCAAGGATACGGCTGGTCAATACGCTACATCTGCTGGGAACTCTGCTTCCGCTGCGCATCAATCTGAGGTAAACGCTGAGAACTCT‑‑GCCACAGCATCCGCTAACTATGCTctgtctcttatt                                                                                                                                                                                          >  1:168610/1‑290 (MQ=255)`  
                                                                                                                   `aCTAAAGACCATGGACCAGAACTCATGGCAAGCACGTAATGAAGCCTTACAGTTCCGTAATGAGGCTGAGACTTTCAGAAACCAAGCGGAGGGCTTTAAGAACGAGTCCAGTACCAACGCTACGAACACAAAGCAGTGGCGCGATGAGACCAAGGGTTTCCGAGACGAAGCCAAGCGGTTCAAGAATACGGCTGGTCAATACGCTACATCTGCTGGGAACTCTGCTTCCGCTGCGCATCAATCTGAGGTAAACGCTGAGAACTAT‑‑GCCACAGCATCCGCTAACTCTGCTCATTTGGCAGaa                                                                                                                                                                                          >  1:641905/1‑301 (MQ=255)`  
                                                                                                                        `aGACCATGGACCAGAACTCATGGCAAGCACGTAATGAAGCCTTACAGTTCCGTAATGAGGCTGAGACTTTCAGAAACCAAGCGGAGGGCTTTAAGATCGAGTCCAGTACCAACGCTACGAACACAAAGCAGTGGCGCGATGAGACCAAGGGTTTCCGAGACGAAGCCAAGCGGTTCAAGAATACGGCTGGTCAATACGCTACATCTGCTGGGAACTCTGCTTCCGCTGCGCATCAATCTGAGGTAAACGCTGAGAACTATGCGCCACAGCATCCGCTAACTATGCCCATTTGGCAGAAccg                                                                                                                                                                                       >  1:456981/1‑299 (MQ=255)`  
                                                                                                                         `gACCATGGACCAGAACTCATGGCAAGCACGTAATGAAGCCTTACAGTTCCGTAATGAGGCTGAGACTTTCAGAAACCAAGCGGAGGGCTTTAAGAACGAGTCCAGTACTAACGCTACGAACACAAAGCAGTGGCGCGATGAGACCAAGGGTTTCCGAGACGAAGCCAAGCGGTTCAAGAATACGGCTGGTCAATACGCTACATCTGCTGGGAACTCTGCTTCCGCTGCGCATCAATCTGAGGTAAACGCTGAGAACTCT‑‑GCCACAGCATCCGCTAACTATGCTCATTTGGCAGAACagcaa                                                                                                                                                                                    >  1:130093/1‑301 (MQ=255)`  
                                                                                                                         `gACCATGGACCAGAACTCATGGCAAGCACGTAATGAAGCCTTACAGTTCCGTAATGAGGCTGAGACTTTCAGAAACCAAGCGGAGGGCTTTAAGAACGAGACCAGTACCAACGCTACGAACACAAAGCAGTGGCGCGATGAGACCAAGGGTTGCCGAGACTAAGACAAGCGGGTCGAGGATACGGCTGGTCAATCCGCTACATCTGTGGGGAACTCTTATTCCGCGGCGCATCACTCTGAGGTAGACGATGAGAACTCT‑‑GCAACAGCATCCGCTAACTTTGCGCATTTGGCTGAACagcaa                                                                                                                                                                                    >  1:636198/1‑301 (MQ=255)`  
                                                                                                                          `aCCATGGACCAGAACTCATGGCAAGCACGTAATGAAGCCTTACAGTTCCGTAATGAGGCTGAGACTTTCAGAAACCAAGCGGAGGGCTTTAAGAACGAGTCCAGTACCAACGCTACGAACACAAAGCAGTGGCGCGATGAGACCAAGGGTTTCCGAGACGAAGCCAAGCGGTTCAAGAATACGGCTGGTCAATACGCTACATCTGCTGGGAACTCTGCTTCCGCTGCGCATCAATCTGAGGTAAACGCTGAGAACTCT‑‑GCCCCAGCATCCGCTAACTATGCTCATTTGGCAGAACagcaaa                                                                                                                                                                                   >  1:100149/1‑300 (MQ=255)`  
                                                                                                                           `acATGGACCAGAAATAAGGGAAAGCAAGGAAGGAAGCCTGAAAGGTCAGTAATGAGGCTGAGACTTTCAGAAACCAAGCGGAGGGCTTTAAGAACGAGTCCAGTACCAACGCTACGAACAAAAAGCAGTGGCGCGATGAGACCAAGGGTTTCCGAGACGAAGCCGAGCGGTTCAAGAATACGGCTGGGCAATACGCTACATCTGCTGGGAACTCTGCTTCCGCTGCGCATCAATCTGAGGTAAACGCTGAGAACTCT‑‑GCCACAGCATCCGCTAACTTTGCTCATTTGGCAGAACagcaagc                                                                                                                                                                                  <  1:117450/300‑1 (MQ=255)`  
                                                                                                                               `gagatgtgtataagagacaGCACGTAATGAAGCCTTACAGTTCCGTAATGAGGCTGAGACTTTCAGAAACCAAGCGGAGGGCTTTAAGAACGAGTCCAGTACCAACGCTACGAACACAAAGCAGTGGCGCGATGAGACCAAGGGTTTCCGAGACGAAGCCAAGCGGTTCAAGAATACGGCTGGTCAATACGCTACATCTGCTGGGAACTCTGCTTCCGCTGCGCATCAATCTGAGGTAAACGCTGAGAACTCT‑‑GCCACAGCATCCGCTAACTTTGCTCATTTGGCAGAACAGCAAGCAGAc                                                                                                                                                                              <  1:602798/283‑1 (MQ=255)`  
                                                                                                                                  `ccAGAACTCATGGCAAGCACGTAATGAAGCCTTACAGTTCCGTAATGAGGCTGAGACTTTCAGAAACCAAGCGGAGGGCTTTAAGAACGAGTCCAGTACCAACGCTACGAACACAAAGCAGTGGCGCGATGAGACCAAGGGTTTCCGAGACGAAGCCAAGCGGTTCAAGGATACGGCTGGTCAATACGCTACATCTGCTGGGAACTCTGCTTCCGCTGCGCATCAATCTGAGGTAAACGCTGAGAACTCT‑‑GCCACAGCATCCGCTAACTATGCTCATTTGGCAGAACAGCAAGCAGAcctg                                                                                                                                                                           >  1:326036/1‑299 (MQ=255)`  
                                                                                                                                             `ggCAAGCACGTAATGAAGCCTTACAGTTCCGTAATGAGGCTGAGACTTTCAGAAACCAAGCGGAGGGCTTTAAGAACGATTCCAGTACCAACGCTACGAACACAAAGCAGTGGCGCGATGAGACCAAGGGTTTCCGAGACGAAGCCAAGCGGTTCAAGAATACGGCTGGTCAATACGCTACATCTGCTGGGAACTCTGCTTCCGCTGCGCATCAATCTGAGGTAAACGCTGAGAACTCT‑‑GCCACAGCATCCGCTAACTATGCTCATTTGGCAGAACAGCAAGCAGACCGTGCgctgtctct                                                                                                                                                                >  1:537282/1‑293 (MQ=255)`  
                                                                                                                                                     `aagagacaggCCTTACAGTTCCGTAATGAGCCTGAGACTTTCAGAAACCAAGCGGAGGGCTTTAAGAACGAGTCCAGTACCAACGCTACGAACACAAAGCAGTGGCGCGATGAGACCAAGGGTTTCCGAGACGAAGCCAAGCGGTTCAAGAATACGGCTGGTCAATACGCTACATCTGCTGGGAACTCTGCTTCCGCTGCGCATCAATCTGAGGTAAACGCTGAGAACTCT‑‑GCCACAGCATCCGCTAACTATGCTCATTTGGCAGAACAGCAAGCAGACCGTGCGGAACGTGAGGCAGACa                                                                                                                                                        <  1:299953/292‑1 (MQ=255)`  
                                                                                                                                                              `gCCTTACAGTTCCGTAATGAGGCTGAGACTTTCAGAAACCAAGCGGAGGGCTTTAAGAACGAGTCCAGTACCAACGCTACGAACACAAAGCAGTGGCGCGATGAGACCACGGGTTTCCGAGACGAAGCCGAGCGGTTCAAGAATACGGCTGGTCAATACGCTACATCTGCTGGGAACTCTGCTTCCGCTGCGCATCAATCTGAGGTAAACGCTGAGAACGCT‑‑GCCACAGCATCCGCTAACTCTGCTCATTTGGCGGAACAGCAAGCAGACCGTGCGGTACGTGAGGCAGACAAGCTGGaaa                                                                                                                                               >  1:79775/1‑301 (MQ=255)`  
                                                                                                                                                              `gCCTTACAGTTCCGTAATGAGGCTGAGACTTTCAGAAACCAAGCGGAGGGCTTTAAGAACGAGTCCAGTACCAACGCTACGAACACAAAGCAGTGGCGCGATGAGACCAAGGGTTTCCGAGACGAAGCCGAGCGGTTCAAGAATACGGCTGGTCAATAAGCTACATCTGCTGGGAACTCTGCTTCCGCTGCGCATCAATCTGAGGTAAACGCTGAGAACTCT‑‑GCAACAGCATCCGCTAACTATGCTCATGTGGCAGAACAGCAAGCAGAACGTGCGGAACGTGAGGCAGACAAGCTGGaaa                                                                                                                                               >  1:221964/1‑301 (MQ=255)`  
                                                                                                                                                                   `aCAGTTCCGTAATGAGGCTGAGACTTTCAGAAACCAAGCGGAGGGCTTTAAGAACGAGTCCAGTACCAACGCTACGAACACAAAGCAGTGGCGCGATGAGACCAAGGGTTTCCGAGACGAAGCCAAGCGGTTCAAGGATACGGCTGGTCAATACGCTACATCTGCTGGGAACTCTGCTTCCGCTGCGCATCAATCTGAGGTAAACGCTGAGAACTCT‑‑GCCACAGCATCCGCTAACTTTGCTCATTTGGCAGAACAGCAAGCAGACCGTGCGGAACGTGAGGCAGACAAGCTGGAAAATTAc                                                                                                                                          <  1:192996/301‑1 (MQ=255)`  
                                                                                                                                                                        `tCCGTAATGAGGCTGAGACTTTCAGAAACCAAGCGGAGGGCTTTAAGAACGAGTCCAGTACCAACGCTACGAACACAAAGCAGTGGCGCGATGAGACCAAGGGTTTCCGAGACGAAGCCAAGCGGTTCAAGGATACGGATGGTCAATACGCTACATCTGCTGGGAACTCTGCTTCCGCTGCGCATCAATCTGAGGTAAATGCTGAGAACTCT‑‑GCCACAGCATCCGCTAAATATGCACATTTGGCAGAACAGTAAGTAGACCGTGCGGAACGTGAGGcctgtctcttatacacatctcatag                                                                                                                                     >  1:143131/1‑277 (MQ=255)`  
                                                                                                                                                                            `tAATGAGGCTGAGACTTTCAGAAACCAAGCGGAGGGCTTTAAGAACGAGTCCAGTACCAACGCTACGAACACAAAGCAGTGGCGCGATGAGACCAAGGGTTTCCGAGACGAAGCCGTGCGGTTCAAGAATACGGCTGGTCAATACGCTACATCTGCTGGGAACTCTGCTTCCGCTGCGCATCAATCTGAGGCAAACGCTGAGAACTCT‑‑GCCACAGCATCCGCTAACTATGCTCATTTGGCAGAACAGCAAGCAGACCGTGCGGAACGTCAGGCAGTCAAGCTGGAAACtttctctgataca                                                                                                                                 >  1:597564/1‑290 (MQ=255)`  
                                                                                                                                                                            `tAATGAGGCTGAGACTTTCAGAAACCAAGCGGAGGGCTTTAAGAACGAGTCCAGTACCAACGCTACGAACACAAAGCAGTGGCGCGATGAGACCAAGGGTTTCCGAGACGAAGCCAAGCGGTTCAAGGATACGGCTGGTCAATACGCTACATCTGCTGGGAACTCTGCTTCCGCTGCGCATCAATCTGAGGTAAACGCTGAGAACTCT‑‑GCCACAGCATCCGCTAACTATGCTCATTTGGCAGAACAGCAAGCAGACCGTGCGGAACGTGAGGCAGACAAGCTAGaaactgtctcttataca                                                                                                                                 >  1:73660/1‑287 (MQ=255)`  
                                                                                                                                                                               `agAGGCTGAGACTTTCAGAAACCAAGCGGAGGGCTTTAAGAACGAGTCCAGTACCAACGCTACGAACACAAAGCAGTGGCGCGATGAGACCAAGGGTTTCCGAGACGAAGCCGAGCGGTTCAAGAATACGGCTGGTCAATACGCTACATCTGCTGGGAACTCTGCTTCCGCTGCGCATCAATCTGAGGTAAACGCTGAGAACGCT‑‑GCCACAGCATCCGCTAACTTTGCTCATTTGGCAGAACAGCAAGCAGACCGTGCGGAACGTGAGGCAGACAAGCTGGAAAATTACAATGGATTGGCt                                                                                                                              <  1:293381/300‑1 (MQ=255)`  
                                                                                                                                                                                  `ggCTGAGACTTTCAGAAACCAAGCGGAGGGCTTTAAGAACGAGTCCAGTACCAACGCTACGAACACAAAGCAGTGGCGCGATGAGACCAAGGGTTTCCGAGACGAAGCCAAGCGGTTCAAGAATACGGCTGGTCAATACGCTACATCTGCTGGGAACTCTGCTTCCGCTGCGCATCAATCTGAGGTAAACGCTGAGAACTCT‑‑GCCACAGCATCCGCTCACTATGCTCATTTGGCAGAACAGCAAGCAGACCGTGCGGAACGTGAGGCAGAAAAGCTGGAAAATTACAAGGGATTGGCtctg                                                                                                                           >  1:444884/1‑298 (MQ=255)`  
                                                                                                                                                                                         `aCTTTCAGAAACCAAGCGGAGGGCTTTAAGAACGAGGCCAGTACCAACGCTACGAAAACAAAGCAGTGGCGCGATGAGACCAAGGGTTTCCGAGACGAAGCCAAGCGGTTCAAGAATACGGCTGGTCAATACGCTACATCTGCTGGGAACTCTGCTTCCGCTGCGCATCAATCTGAGGTAAACGCTGAGAACTCT‑‑GCCACAGCATCCGCTAACTATGCTCATTTGGCAGAACAGCAAGCAGACCGTGCGGAACGTGAGGCAGACAAGCTGGAAAATTACAATGGATTGGCTGGTGCAATTg                                                                                                                    <  1:276289/301‑1 (MQ=255)`  
                                                                                                                                                                                            `ttCAGAAACCAAGCGGAGGGCTTTAAGAACGAGTCCAGTACCAACGCTACGAACACAAAGCAGTGGCGAGATGAGACCAAGGGTTTCCGAGACGAAGCCAAGCGGTTCAAGGATACGGCTGGTCAATACGCTACATCTGCTGGGAACTCTGCTTCCGCTGCGCATCAATCTGAGGTAAACGATGAGAACTCT‑‑GCCACAGCATCCGCTAACTTTGCTCGTTTGGCAGAACAGCAAGCAGACCGTGCGGAACGTGAGGCAGACAAGCTGGAAAATTACAATGGATTGGCTGGTGCAATTGATa                                                                                                                 <  1:490950/301‑1 (MQ=255)`  
                                                                                                                                                                                               `ggtataagagacaggggCTTTAAGAACGAGTCCAGTATCAACGCTACGAACACAAAGCAGTGGCGCGATGAGACCAAGGGTTTCCGAGACGAAGGCAAGCGGTTCAAGGATACGGCTGGTCAATACGCTACATCTGCTGGGAACTCTGCTTCCGCTGCGCATCAATCTGAGGTAAACGCTGAGAACTCT‑‑GCCACAGCATCCGCTAACTCTGCTCATTTGGCAGAACAGCAAGCAGACCGTGCGGCACGTGAGGCAGACAAGCTGGAAAATTACAATGGATTGGCTGGTGCAATTGATAAgg                                                                                                              <  1:593017/287‑1 (MQ=255)`  
                                                                                                                                                                                                `aaaaCCAAGCGGTGGGCTTTAAGAACGATTCCAGTACAAACGCTACGAACACAAAGAAGTGGCGCGATGAGACCAAAGGTTTCCGAGACGAAGTCGAGCGGTTCAAGAATACGGCTGGTCAATACGCTAAATCTGCTAGGAACTCTGCTTCCGATGCGCATCAATCTGAGGTAAACGCTAAGAACTCT‑‑GCCACAGCATCCGCTAACTTAGCTCATTTGGCAGAACAGCAAGCAGACCGTGCGGAACGTGAGGCAGACAAGCTGGAAAATTACAATGGATTGGCTGGTGCAATTGATAAGGt                                                                                                             <  1:4133/300‑1 (MQ=255)`  
                                                                                                                                                                                                      `aaGCGGGTGGCTTTAAGAACTAGTCAAGTACCAACGATACGAACACAAAGCAGTGGCGCGATGAGACCAAGGGTTTCCGAGACGAAGCCAAGCGGTTCAAGGATACGGCTGGTCAATACGCTACATCTGCTGGGAACTCTGCTTCCGCTGAGCATCAATCGGAGGTAAACGCTGAGAACTCT‑‑GCCACAGCATCCGCTAACTTTGCTCATTTGGCAGAACAGCAAGCAGACCGTGCGGAACGTGAGGCAGACAAGCTGGAAAATTACAATGGATTGGCTGGTGCAATTGATAAGGTAGATgg                                                                                                       <  1:149127/301‑1 (MQ=255)`  
                                                                                                                                                                                                        `gCGGAGGGCTTTAAGAACGAGTCCAGTACCAACGCTACGAACACAAAGCAGTGGCGCGATAAGACCAAGGGTTTCCGAGACGAAGCCAAGCGGTTCAAGGATACGGCTGGTCAATACGCTACATCTGCTGGGAACTCTGCTTCCGCTGCGCATCAATCTGAGGTAAACGCTGAGAACTCT‑‑GCCACAGCATCCGCTAACTTTGCTCATTTGGCATAACAGCAAGCAGACCGTGCGGAACGTGAGGCAGACAAGCTGGACAATTACAATGGATTGCCTGGTTCAATTGATAAGGTAGCTGGaa                                                                                                     >  1:370373/1‑301 (MQ=255)`  
                                                                                                                                                                                                         `ggatgtgtataagagacaggTCCAGTACCAACGCTACGAACACAAAGCAGTGGCGCGATGAGACCAAGGGTTTCCGAGACGAAGCCAAGCGGTTCAAGGATACGGCTGGTCAATACGCTACATCTGCTGGGAACTCTGCTTCCGCTGCGCATCAATCTGAGGTAAACGCTGAGAACTCT‑‑GCCACAGCATCCGCTAACTATGCTCATTTGGCAGAACAGCAAGCAGACCGTGCGGAACGTGAGGCAGACAAGCTGGAAAATTACAATGGATTGGCTGGTGCAATTGATAAGGTAGATGGAAc                                                                                                    <  1:16273/282‑1 (MQ=255)`  
                                                                                                                                                                                                              `ggCTTTAAGAACGAGTCCAGTACCAACGCTACGAACACAAAGCAGTGGCGCGATGAGACCAAGGGTTTCCGAGACGAAGCAAAGCGGTTCAAGAATACGGCTGGTCAATACGCTACATCTGCTGGGAACTCTGCTTCCGCTGCGCATCAATCTGAGGTAAACGTTGAGAACTCT‑‑GCCACAGCATCCGCTAACTATGCTCATTTGGCAGAACAGCAAGCAGACCGTGCGGAACGTGAGGCAGCCAAGCTGGAAAATTACAATGGATTGGCTGGTGCAATTGATAAGGTAGATGGAACCAAtg                                                                                               <  1:282230/301‑1 (MQ=255)`  
                                                                                                                                                                                                               `gCTTTAAGAACGAGTCCAGTACCAACGCTACGAACACAAAGCAGTGGCGCGATGAGACCAAGGGTTTCCGAGACGAAGCCAAGCGGTTCAAGAATACGGCTGGTCAATACGCTACATCTGCTGGGAACTCTGCTTCCGCTGCGCATCAATCTGAGGTAAACGCTGAGAACTCT‑‑GCCACAGCATCCGCTAACTATGCTCATTTGGCAGAACAGCAAGCAGACCGTGCGGAACGTGAGGCAGACAAGCTGGAAAATTACAATGGATTGGCTGGTGCAATTGATAAGGTAGATGGaaacctgtc                                                                                              >  1:239679/1‑294 (MQ=255)`  
                                                                                                                                                                                                               `gCTTTAAGAACGAGTCCAGTACCAACGCTACGAACACAAAGCAGTGGCGCGATGAGACCAAGGGTTTCCGAGACGAAGCCAAGCGGTTCAAGAATACGGCTGGTCAATACGCTACATCTGCTGGGAACTCTGCTTCCGCTGCGCATCAATCTGAGGTAAACGCTGAGAACTCT‑‑GCCACAGCATCCGCTAACTATGCTCATTTGGCAGAACAGCAAGCAGACCGTGCGGAACGTGAGGCAGACAAGCTGGAAAATTACAATGGATTGGCTGGTGCAATTGATAAGGTAGATGGAACCAAtgt                                                                                              >  1:58314/1‑301 (MQ=255)`  
                                                                                                                                                                                                                   `taataaCGAGTCCAGTACCAACGCTACGAACACAAAGCAGTGGCGCGATGAGACCAAGGGTTTCCGAGACGAAGACAAGCGGTTCAAGGATACGGCTGGTCAATACGCTACATCTGCTGGGAACTCTGCTTCCGCTGCGCATCAATCTGAGGTAAACGCTGAGAACTCT‑‑GCCACAGCATCCGCTAACTTTGCTCATTTGGCAGAACAGCAAGCAGACCGTGCGGAACGTGAGGCAGACAAGCTGGAAAATTACAATGGATTGGCTGGTGCAATTGATAAGGTAGATGGAACCAATGTGTAc                                                                                          <  1:336322/301‑1 (MQ=255)`  
                                                                                                                                                                                                                   `tAAGAACGAGTCCAGTACCAACGCTACGAACACAAAGCAGTGGCGCGATGAGACCAAGGGTTTCCGAGACGAAGCCAAGCGGTTCAAGGATACGGCTGGTCAATACGCTACATCTGCTGGGAACTCTGCTTCCGCTGCGCATCAATCTGAGGTAAACGCTGAGAACTCT‑‑GCCACAGCATCCGCTAACTATGCTCATTTGGCAGAACAGCAAGCAGACCGTGCGGAACGTGAGGCAGACAAGCTGGAAAATTACAATGGATTGGCTGGTGCAATTGATAAGGTAGATGGAACCAATGTGTAc                                                                                          <  1:510414/301‑1 (MQ=255)`  
                                                                                                                                                                                                                        `gtgtataagagacaggcGCTACGAACACAAAGCAGTGGCGCGATGAGACCAAGGGTTTCCGAGACGAAGCCAAGCTGTTCAAGGATACGGCTGGTCAATACGCTACATCTGCTGGGAACTCTGCTTCCGCTGCGCATCAATCTGAGGTAAACGCTGAGAACTCT‑‑GCCACAGCATCCGCTAACTATGCTCATTTGGCAGAACAGCAAGCAGACCGTGCGGAACGTGAGGCAGACAAGCTGGAAAATTACAATGGATTGGCTGGTGCAATTGATAAGGTAGATGGAACCAATGTGTACTggaa                                                                                     <  1:24682/285‑1 (MQ=255)`  
                                                                                                                                                                                                                        `aCGAGTCCAGTACCAACGCTAAGAACACAAAGCAGTGGCGCGATGAGACCAAGGGTTTCCGAGACGAAGCCAAGCGGTTCAAGGATACGGCTGGTCAATACGCTACATCTGCTGGGAACTCTGCTTCCGCTGCGCATCAATCTGAGGTAAACGCTGAGAACTCT‑‑GCCACAGCATCCGCTAACTTTGCTCATTTGGCAGAACAGCAAGCAGACCGTGCGGAACGTGAGGCAGACAAGCTGGAAAATTACAATGGATTGGCTGGTGCAATTGATAAGGTAGATGGAACCAATGTGTACTggaa                                                                                     <  1:516177/301‑1 (MQ=255)`  
                                                                                                                                                                                                                        `aCGAGTCCAGTACAAACGATACAAACACAAAGCAGTGGCGCGATGAGACCAAGGGTTTCCGAGACGAAGCCAAGCGGTTCAAGTATACGGCAGGTCAATACACTACATCGGCTAGGAACTCTGCTTCCGCTGCGCATCAATCTGAGGTAAACGCTAAGAACTCT‑‑GCCACAGCATCCGCAAACTCTGCTCATTTGGCAGAACAGCAAGCAGACCGTGCGGAACGTGAGGCAGACAAGCTGGAAAATTACAATGGAGTGGCTGGTGCAATTGATAAGGTAGATGGAACCAATGTGTACTggaa                                                                                     <  1:252279/301‑1 (MQ=255)`  
                                                                                                                                                                                                                                 `gTATCAACGCTACGAACACAAAGCAGTGGCGCGATGAGACCAAGGGTTTCCGAGACGAAGCCAAGCTGTTCAGGGATACGGCTGGTCAATACGCTACATCTGCTGGGAACTCTGCTTCCGCTGCGCATCAATCTGAGGTAAACGCTTAGAACTCT‑‑GCCACAGCATCCGCTAACTCTGCTCATTTGGCAGAACAGCAAGCAGACCGTGCGGCACGTGAGGCAGACAAGCTGGAAAATTACAATGGATTGGCTGGTGCAATTGATAAGGTAGATGGAACCAATGTGTACTGGAAAGGAAatat                                                                            >  1:313115/1‑301 (MQ=255)`  
                                                                                                                                                                                                                                 `gTACCAACGCTACGAACACAAAGCAGTGGCGCGATGAGACCAAGGGTTTCCGAGACGAAGCCGAGCGGTTCAAGAATACGGCTGGTCAATACGCTACATCTGCTGGGAACTCTGCTTCCGCTGCGCATCAATCTGAGGTAAACGCTGAGAACTCT‑‑GCCACAGCAGCCGCTAACTCTGCTCATTTGGCAGAACAGCAAGCAGACCGTGCGTAACGTGAGGCAGACAAGCTTGAAAATTACAATGGATTGGCTGGTGCAATTGATAAGGTAGATGGAACCAATGTGTACTGGAAAGGAAatat                                                                            >  1:666357/1‑301 (MQ=255)`  
                                                                                                                                                                                                                                 `gTACCAACGCTACGAACACAAAGCAGTGGCGCGATGAGACCAAGGGTTTCCGAGACGAAGCCAAGCGGTTCAAGGATACGGCTGGTCAATACGCTACATCTGCTGGGAACTCTGCTTCCGCTGCGCATCAATCTTAGGTAAACGCTGAGAACTAT‑‑GCCACAGCATCCGCTAACTCTGCTCATTTGGCAGAACAGCAAGCAGACCGTGCGGAACGTGAGGCAGACAAGCTGGAAAATTACAATGGATTGGCTGGTGCAATTGATAAGGTAGATGGAACCAATGTGTACTggaaaggctgtct                                                                            >  1:660583/1‑295 (MQ=255)`  
                                                                                                                                                                                                                                 `gTACCAACGCTACGAACAAAACGAAGGGGCGCGATGAGACCAAGGGTTTCCGAGACGAATCCGAGCGGTTCAAGAATACGGTTGGTAAATACGCTACATCTGCTGGGAACTCTGCTTCCGCTGCGCATCAATCTGAGGTAAACGATGAGAACGCT‑‑GCCACAGCATCCTCTAACTCTGCTCATTTGGCAGAACAGCAAGCAGACCGTGCGGAACGTGAGGCAGACAAGCTGGAAAATTACAATGGATTGGCTGGTGCAATTGATAAGGTAGATGGAACCAATGTGTACTGGAAAGGAAatat                                                                            <  1:432811/301‑1 (MQ=255)`  
                                                                                                                                                                                                                                          `cTACGAACACAAAGCAGTGGCGCGATGAGACCAAGGGTTTCCGAGACGAAGCCAAGCGGTTCAAGGATACGGCTGGTCAATACGCTACATCTGCTGGGAACTCTGCTTCCGCTGCGCATCAATCTGAGGTAAACGCTGAGAACTCT‑‑GCCACAGCATCCGCTAACTATGCTCATTTGGCAGAACAGCAAGCAGACCGTGCGGAACGTGAGGCAGACAAGCTGGAAAATTACAATGGATTGGCTGGTGCAATTGATAAGGTAGATGGAACCAATGTGTACTGGAAAGGAAATATTcctgactc                                                                   >  1:320040/1‑294 (MQ=255)`  
                                                                                                                                                                                                                                           `tACGAACACAAAGCAGTGGCGCGATGAGACCAAGGGTTTCCGAGCCGAAGCCAAGCGGTTCAAGAATACGGCTGGTCAATACGCTACATCTGCTGGGAACTCTGCTTCCGCTGCGCATCAATCTGAGGTAAACGCTGAGAACTCT‑‑GCCACAGCATCCGCTAACTTTGCTCATTTGGCAGAACAGCAAGCAGACCGTGCGGAACGTGAGGCAGACAAGCTGGAAAATTACAATGGATTGGCTGGTGCAATTGATAAGGTAGATGGAACCAATGTGTACTGGAAAGGAAATATTCCCGCTAAc                                                                  >  1:135429/1‑301 (MQ=255)`  
                                                                                                                                                                                                                                           `tACGAACACAAAGCAGTGGCGCGATGAGACCAAGGGTTTCCGAGACGAAGCCGAGCGGTTCAAGAATACGGCTGGTCAATACGCTACATCTGCTGGGAACTCTGCTTCCGCTGCGCATCAATCTGAGGTAAACGCTGAGAACTCT‑‑GCCACAGCAGCCGCTAACTCTGCTCATTTGGCAGAACAGCAAGCAGACCGTGCGGAACGTGAGGCAGACAAGCTGGAAAATTACAATGGATTGGCTGGTGCAATTGATAAGGTAGATGGAACCAATGTGTACTGGAAAGGAAATATTCACGCTaaa                                                                  >  1:64843/1‑300 (MQ=255)`  
                                                                                                                                                                                                                                                       `gCAGTGGCGCGATGAGACCAAGGGTTTCCGAGACGAAGCCAAGCGGTTCAAGGATACGGCTGGTCAATACGCTACATCTGCTGGGAACGCTGCTTCCGCTGCGCATCAATCTGAGGTAAACGCTGAGAACTCT‑‑GCCACAGCATCCGCTAACTATGCTCATTTGGCAGAACAGCAAGCAGACCGTGAGGAACGTGAGGCAGACCAGCTGGAAAATAACCATGGATTGGCTGGTGCAATTGGTAAGGTAGATGGAACCCATGTGTACTGGACAGGACATATTCACGCTAACGGGCTCCTTTAc                                                      >  1:258443/1‑301 (MQ=255)`  
                                                                                                                                                                                                                                                        `gaGTGGCGGGATGGGACCAAGGGTGGCCGAGACGAAGACAAGCTGTTCAAAGAGACGGCGTGTCAATACGCGACATCTGCTGGGAAATCTGCTTCCGCTGCGCATCAATCTGAGGTAAACGGTGAGAAATCT‑‑GCAACCGCAGCCGCTAACTGTGCTAATGTGGCAGAACAGCAAGCAGACCGTGCGGAACGTGAGGCAGACAAGCTGGAGAAGTACAATGGATTGGATGGGGGAATTGATAAGGTAGATGGAAGCAATAAGTACTGGAAAGGAAATATTCACGCTAACGGGCGCCTTTACa                                                     <  1:215323/300‑1 (MQ=255)`  
                                                                                                                                                                                                                                                            `gacaggATGAGACCAAGGGTATCCGAGACGAAGACAAGCGGTTCAAGAATACGGCTGGTCAATACGCTACATCTGCTGGGAACTCGGCTTCCGCTGCGCATCAATCTGAGGTAAACGCTGAGAACTCT‑‑GCCACAGCATCCGCTAACTATGCTCATTTGGAAGAACAGCAAGCAGACCGTGCGGAACGTGAGGCAGACAAGCTGGAAAATTACAATGGATTGGCTGGTGGAATTGATAAGGTAGATGGAACCAATGTGTACTGGAAAGGAAATATTCACGCTAACGGGCGCCTTTACAGGAc                                                 <  1:93136/296‑1 (MQ=255)`  
                                                                                                                                                                                                                                                             `gctctaTGAGACCAAGGGTTTCCGAGACGAAGCCAAGCTGTTAAAGGATACGGCTGGTCAATACGCTACATCTGCTGGTAACTCTGCTTCCGCTTCTCATCAATCTGAGGTAAACGCTGAGAACTCT‑‑GCCACAGCATCCGCTAACTTTGCTCATTTGGCAGAACAGCAAGCAGACCGTGCGGAACGTGAGGCAGACAAGCTGGAAAATTACAATGGATTGGCTGGTGCAATTGATAAGGTAGATGGAACCAATGTGTACTGGAAAGGAAATATTCACGCTAACGGGCGCCTTTACATGAcc                                                <  1:52105/296‑1 (MQ=255)`  
                                                                                                                                                                                                                                                                   `ggagaCCAAGGGTTTCCGAGACGAAGCCAAGCGGTTCAAGGATACGGCTGGTCAATACGCTACATCTGCTGGGAACTCTGCTTCCGCTGCGCATCAATCTGAGGTAAACGCTGAGAACTCT‑‑GCCACAGCATCCGCTAACTATGCTCATTTGGCAGAACAGCAAGCAGACCGTGCGGAACGTGAGGCAGACAAGCTGGAAAATTACAATGGATTGGCTGGTGCAATTGATAAGGTAGATGGAACCAATGTGTACTGGAAAGGAAATATTCACGCTAACGGGCGCCTTTACATGACCACAAAc                                          <  1:228459/300‑1 (MQ=255)`  
                                                                                                                                                                                                                                                                             `ggTTTCCGAGACGAAGCCAAGCGGTTCAAGGATACGGCTGGTCAATACGCTACATCTGCTGGGACCTCTGCTTCCGCTGCGCATCAATCTGAGGTAAACGCTGAGAACTCT‑‑GCCACAGCATCCGCTAACTATGCTCATTTGGCAGAACAGCAAGCAGACCGTGCGGAACGTGAGGCAGACAAGCTGGAAAATTACAATGGATTGGCTGGTGCAATTGATAAGGTAGATGGAACCCATGTGTACTGGAAAGGAAATATTCACGCTAACGGGCGCCTTTACATGACCACAAACGGTTTTGACt                                >  1:307079/1‑301 (MQ=255)`  
                                                                                                                                                                                                                                                                                        `cGAATCCAAGCGGTTCAAGAATACGGCTGGTCAATACGCTACATCTGCTGGGAACTCTGCTTCCGCTGCGCATCAATCTGAGGTAAACGCTGAGAACTCT‑‑GCCACAGCATCCGCTAACTATGCTCATTTGGCAGAACAGCAAGCAGACCGTGCGGAACGTGAGGAAGACAAGCTGGAAAATTACAATGGATTGGCTGGTGCAATTGATAAGGTAGATGGAACCAATGTGTACTGGAAAGGAAATATTCACGCTAACGGGCGCCTTTACATGACCACAAACGGTTTTGACTGTGGCCAGTAt                     <  1:264587/301‑1 (MQ=255)`  
                                                                                                                                                                                                                                                                                        `agAAGCCAAGCGGTTAAAGAATACGGCTGGTCAATACGCTACATCTGCTGGGAACTCTGCTTCCGCTGCGCATCAATCTGAGGTAAACGCTGAGAACTCT‑‑GCCACAGCATCCGCTAACTATGCTCATTTGGCAGAACAGCAAGCAGACCGTGCGGAACGTGAGGCAGACAAGCTGGAAAATTACAATGGATTGGCTGGTGCAATTGATAAGGTAGATGGAACCAATGTGTACTGGAAAGGAAATATTCACGCTAACGGGCGCCTTTACATGACCACAAACGGTTTTGACTGTGGCCAGTAt                     <  1:549649/300‑1 (MQ=255)`  
                                                                                                                                                                                                                                                                                              `cAAGCTGTTCAAGAATACAGACGGTCAATACCCTACATCTGATGGGAACTCTGCTGTCGCGGCGAAGTAATCTGAGGTAAACGCTGAGAACTCC‑‑GCCACAGCATCCGCGAACTATGCTCAATTGGCAGAACAGCAAGCAGACCGTGCGGAACGTGATGCAGACAAGCTGGAAAATTACAATGGATTGGCTGGTTCAATTGATAAGGTAGATGGAACCAATGTGTACTGGAAAGGAAATATTCACTCTAACGGGCGCCTTTACATGACCACAAACGGTTTTGACTGTGGCCAGTATCAACAg               <  1:105944/301‑1 (MQ=255)`  
                                                                                                                                                                                                                                                                                                    `gTTCAAGAATACGGCTGGTCAATACGCTACATCTGCTGGGAACTCTGCTTCCGCTGCGCATCAATCTGAGGTAAACGCTGAGAACTCT‑‑GCCACAGCATCCGCTAACTATGCTCATTTGGCAGAACAGCAAGCAGACCGTGCGGAACGTGAGGCAGACAAGCTGGAAAATTACAATGGATTGGCTGGTGCAATTGATAAGGTAGATGGAACCAATGTGTACTGGAAAGGAAATATTCACGCTAACGGGCGCCTTTACATGACCACAAACGGTTTTGACTGTGGCCAGTATCAACAGTTCttt         >  1:375985/1‑301 (MQ=255)`  
                                                                                                                                                                                                                                                                                                    `gTTCAAGAATACGGCTGGTCAATACGCTACATCTGCTGGGAACTCTGCTTCCGCTGCGCATCAATCTGAGGTAAACGCTGAGAACTCT‑‑GCCACAGCATCCGCTAACTATGCTCATTTGGCAGAACAGCAAGCAGACCGTGCGGAACGTGAGGCAGACAAGCTGGAAAATTACAAAGGATTGGCTGGTGCAATTGATAAGGTAGATGGAACCAATGTGTACTGGAAAGGAAATATTCACGCTAACGGGCGCCTTTACATGACCACAAACGGTTTTGACTGTGGCCAGTATCAACAGTTCttt         >  1:497406/1‑301 (MQ=255)`  
                                                                                                                                                                                                                                                                                                      `taaaGGATACGGCTGGTCAATACGCTACATCTGCTGGGAACTCTGCTTCCGCTGCGCATCAATCTGAGGTAAACGCTGAGAACTCT‑‑GCCACAGCATCCGCTAACTATGCTCATTTGGCAGAACAGCAAGCAGACCGTGCGGAACGTGAGGCAGACAAGCTGGAAAATTACAATGGATTGGCTGGTGCAATTGATAAGGTAGATGGAACCAATGTGTACTGGAAAGGAAATATTCACGCTAACGGGCGCCTTTACATGACCACAAACGGTTTTGACTGTGGCCAGTATCAACAGTTCTTtgg       <  1:39384/299‑1 (MQ=255)`  
                                                                                                                                                                                                                                                                                                          `gAATACGGCTGGTCAATACGCTACATCTGCTGGGAACTCTGCTTCCGCTGCGCATCAATCTGAGGTAAACGCTGAGAACTCT‑‑GCCACAGCATCCGCTAACTATGTTCATTTTGCAGAACAGCAAGCAGACCGTGCGGAACGTGTGGCAGACAAGCTGGAAAATTACAATGGATTGGCTGGTGCAATTGATAAGGTAGATGGAACCAATGTGTACTGGAAAGGAAATATTCATGCTAACGGGCGCCTTTAATTGATCACAAACGGTTTTGACTGTTGCCAGTATCAACAGGTCTTtggtggg   >  1:387387/1‑300 (MQ=255)`  
                                                                                                                                                                                                                                                                                                          `gAATACGGCTGGTCAATACGCTACATCTGCTGGGAACTCTGCTTCCGCTGCGCATCAATCTGAGGTAAACGCTGAGAACTCT‑‑GCCACAGCATCCGCTAACTATGCTCATTTGGCAGAACAGCAAGCAGACCGTGCGGAACGTGAGGCAGACAAGCTGGAAAATTACAATGGATTGGCTGGTGCAATTGATAAGGTAGATGGAACCAATGTGTACTGGAAAGGAAATATTCACGCTAACGGGCGCCTTTACATGACCACAAACGGTTTTGAcctgtctcttatacacatctccgagcccacg   >  1:467924/1‑271 (MQ=255)`  
                                                                                                                                                                                                                                                                                                          `gAATACGGCTGGTCAATACGCTACATCTGCTGGGAACTCTGCTTCCGCTGCGCATCAATCTGAGGTAAACGCTGAGAACTCT‑‑GCCACAGCATCCGCTAACTATGCTCATTTGGCAGAACAGCAAGCAGACCGTGCGGAACGTGAGGCAGACAAGCTGGAAAATTACAATGGATTGGCTGGTGCAATTGATAAGGTAGATGGAACCAATGTGTACTGGAAAGGAAATATTCACGCTAACGGGCGCCTTTACATGACCACAAACGGTTTTGACTGTGGCCAGTATCAACAGTTCTTTggtggt   >  1:234724/1‑301 (MQ=255)`  

                                                                                                                                                                                                                                                                                                           `|`                                                                                                                                                                                                                                                                                                                
`ACCTCACTACGGATACTATCGGTGTCAATAACGATGGTCACTTGGATGCTCGTGGTCGTCGAATTGTGAACCTAGCGAACGCCGTGGATGACCGCGATGCTGTTCCGTTTGGTCAACTAAAGACCATGAACCAGAACTCATGGCAAGCACGTAATGAAGCCTTACAGTTCCGTAATGAGGCTGAGACTTTCAGAAACCAAGCGGAGGGCTTTAAGAACGAGTCCAGTACCAACGCTACGAACACAAAGCAGTGGCGCGATGAGACCAAGGGTTTCCGAGACGAAGCCAAGCGGTTCAAGAATACGGCTGGTCAATACGCTACATCTGCTGGGAACTCTGCTTCCGCTGCGCATCAATCTGAGGTAAACGCTGAGAACTCT‑‑GCCACAGCATCCGCTAACTCTGCTCATTTGGCAGAACAGCAAGCAGACCGTGCGGAACGTGAGGCAGACAAGCTGGAAAATTACAATGGATTGGCTGGTGCAATTGATAAGGTAGATGGAACCAATGTGTACTGGAAAGGAAATATTCACGCTAACGGGCGCCTTTACATGACCACAAACGGTTTTGACTGTGGCCAGTATCAACAGTTCTTTGGTGGTG  >  NC_001604.1/34967‑35566`

  

Alignment Legend

Aligned base mismatch/match (shaded by quality score): `ATCG/ATCG` `< 3 ≤` `ATCG/ATCG` `< 10 ≤` `ATCG/ATCG` `< 20 ≤` `ATCG/ATCG` `< 34 ≤` `ATCG/ATCG` `< 38 ≤` `ATCG/ATCG`

Unaligned base: `atcg`    Masked matching base: `atcg`    Alignment gap: `‑`    Deleted base: `‑`
